# Inter-annual maintenance of the fine-scale genetic structure in a biennial plant

**DOI:** 10.1038/srep37712

**Published:** 2016-11-24

**Authors:** Javier Valverde, José María Gómez, Cristina García, Timothy F. Sharbel, María Noelia Jiménez, Francisco Perfectti

**Affiliations:** 1 Departamento de Ecología, Universidad de Granada, E-18071, Granada, Spain; 2 Departamento de Ecología Funcional y Evolutiva, Estación Experimental de Zonas Áridas (EEZA-CSIC), E-04120, Almería, Spain; 3 Plant Biology, CIBIO/InBio, Centro de Investigação em Biodiversidade e Recursos Genéticos, Laboratório Associado, Universidade do Porto. Campus Agrário de Vairão, 4485-661, Vairão, Portugal; 4 Leibniz Institute for Plant Genetics and Crop Plant Research (IPK), 06466, Gatersleben, Germany; 5 Global Institute for Food Security (GIFS), University of Saskatchewan, Saskatoon, Canada; 6 Departamento de Botánica, Universidad de Granada, E-18071, Granada, Spain; 7 Departamento de Genética, Universidad de Granada, E-18071, Granada, Spain

## Abstract

Within plant populations, space-restricted gene movement, together with environmental heterogeneity, can result in a spatial variation in gene frequencies. In biennial plants, inter-annual flowering migrants can homogenize gene frequencies between consecutive cohorts. However, the actual impact of these migrants on spatial genetic variation remains unexplored. Here, we used 10 nuclear microsatellite and one plastid genetic marker to characterize the spatial genetic structure within two consecutive cohorts in a population of the biennial plant *Erysimum mediohispanicum* (Brassicaceae). We explored the maintenance of this structure between consecutive flowering cohorts at different levels of complexity, and investigated landscape effects on gene flow. We found that cohorts were not genetically differentiated and showed a spatial genetic structure defined by a negative genetic-spatial correlation at fine scale that varied in intensity with compass directions. This spatial genetic structure was maintained when comparing plants from different cohorts. Additionally, genotypes were consistently associated with environmental factors such as light availability and soil composition, but to a lesser extent compared with the spatial autocorrelation. We conclude that inter-annual migrants, in combination with limited seed dispersal and environmental heterogeneity, play a major role in shaping and maintaining the spatial genetic structure among cohorts in this biennial plant.

Spatial Genetic Structure (SGS) is the non-random spatial distribution of genotypes, a pattern occurring in most plant populations^1^. It results from an equilibrium among space-restricted gene movement, genetic drift and local selection^2^,^3^. SGS is most of the times the result of isolation by distance (IBD)^2^, in which local mating or short-distance seed dispersal generates a pattern such that kinship among individuals decreases with distance. The scale at which these processes act varies among species^1^, and recently, several studies have demonstrated that it can also happen at very fine scales^4^,^5^,^6^,^7^. The extent and strength of SGS depends on seed and pollen dispersal strategies as well as on mating system^1^,^8^,^9^. Moreover, dispersal can be directionally biased, resulting in differences in IBD intensity at different spatial directions^10^,^11^,^12^,^13^ that promote spatial asymmetric SGS, a pattern also occurring at fine-scales^5^. This spatial asymmetry usually responds to environmental heterogeneity^10^,^11^. At-site variability in ecological conditions can create differences in habitat suitability and, therefore, affect genetic connectivity via seed or pollen dispersal^12^,^14^. Regarding the latter, an increasing number of studies are analysing environmental heterogeneity to understand fine-scale SGS and its effects on population dynamics and evolution.

Studies dealing with the temporal scale of SGS have been mostly aimed at disentangling how demography affects SGS by comparing among age-classes^15^,^16^,^17^,^18^. In perennials, age-classes coexist in the same spatio-temporal context, implying that individuals initially from different cohorts classes may interbreed. In contrast, in strict biennials, i.e., those plants completing their life cycle in two years, individuals plants from consecutive cohorts are reproductively isolated because they do not overlap in their flowering year^19^. However, this presumption is far from reality because only a few species are strictly biennial^20^,^21^,^22^. Several processes promote variation in biennial life span, permitting the existence of inter-annual migrants that bridge years (‘facultative biennials’)^23^,^24^. These inter-annual migrants favour gene flow between cohorts, leading to homogenisation of their allelic frequencies^25^. However, little is known regarding how inter-annual migrants affect the similarities in SGS among cohorts of a biennial, particularly for small plant populations where temporal changes in population size could dramatically vary their genetic composition^26^. We think that the spatial location at which inter-annual migrants arrive to the next cohort may affect SGS. Particularly, in plants presenting limited seed dispersal, the localized arrival of inter-annual migrants may have predictable effects on the SGS, equalizing the spatial variation in allelic frequencies between consecutive cohorts and balancing population size fluctuations.

We have studied two consecutive cohorts of the biennial plant *Erysimum mediohispanicum* (Brassicaceae) to explore fine-scale SGS and to investigate the temporal maintenance of SGS between cohorts. *E. mediohispanicum* is endemic to the Iberian Peninsula^27^, where a highly diverse assemblage of pollinators visits their flowers. Although slightly self-compatible, this species needs pollinators for a complete seed set^28^, whose dispersal occurs at very short distances due to its barochorous dispersal strategy^29^. Populations of this species are patchily distributed and formed by tens to several hundreds of individuals^30^. These populations vary largely in size due to climate fluctuations, especially linked to drought years.

We have analysed the temporal maintenance of SGS between cohorts of *E. mediohispanicum* using 10 nuclear microsatellite and one plastid DNA marker. We also assess to what extent spatial environmental variation shapes the observed SGS. We first explore if cohorts were genetically differentiated, followed by characterization and comparison of SGS between cohorts at different levels: (1) by using spatial principal component analysis (sPCA) to assess global genetic structure; (2) by testing for isotropic SGS using kinship-distance distograms; (3) by using bearing correlograms to visualize SGS asymmetry (anisotropy). Finally, we assess associations among the spatial variation in genotypes and environmental factors using autoregressive spatial models.

## Results

### Genetic characterization

**Figure 1:**
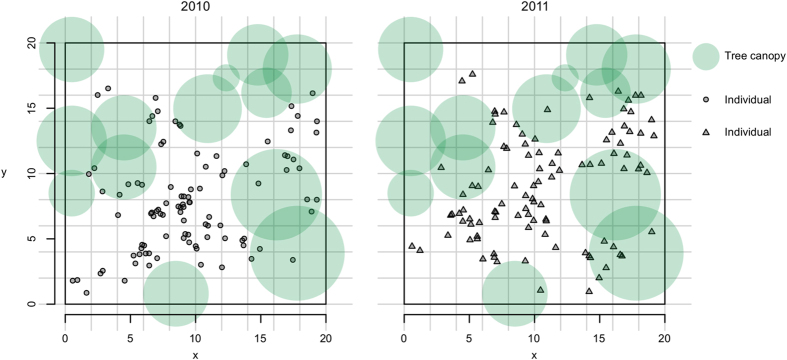
Spatial location of the marked plants. Tree canopy is represented with green circles to evidence the fine-scale environmental heterogeneity. Scale is in meters.

We successfully characterized 200 sampled individuals (100 per cohort, Fig. 1). The highest proportion of missing data (3%) was found in the locus E3 in 2011. Allelic richness was 11.7, ranging between four and 22 per locus (Table 1). Most loci were at Hardy-Weinberg equilibrium, with only one locus in 2010 and two in 2011 presenting a deficit of heterozygotes. The analysis of the plastid *trnL-trnF* spacer yielded five haplotypes with an uneven occurrence across the population, with 95.5% of individuals bearing one of the two most frequent haplotypes.

### Inter-annual genetic structure

All loci exhibited extremely low values of the genetic variance component related to inter-annual differences (*F*_*ST*_ < 0.006, p > 0.05 for all loci), similar to what we found for the multilocus value (*F*_*ST*_ < 0.001, p = 0.226). These low values were consistent with the lack of significance found after an AMOVA (σ^2^ = 0; p = 0.765). Moreover, the proportion of inferred migrants between cohorts was high (0.272).

### Spatial variation of allele frequencies

**Figure 2:**
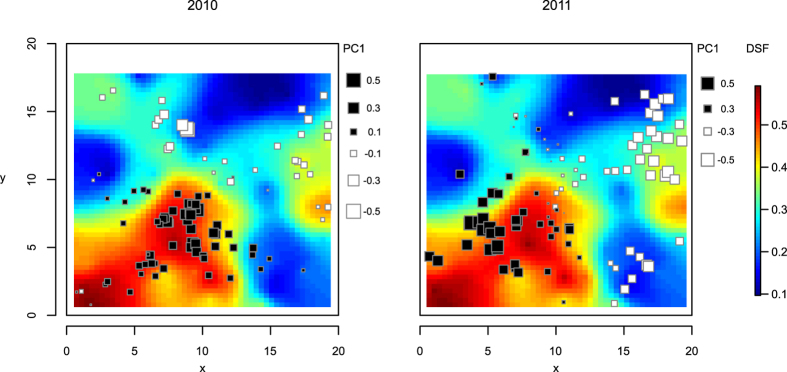
Spatial representation of the first vector from the sPCA for both cohorts and light availability (DSF). Individuals tend to be surrounded by other individuals with similar score values, indicating local aggregation of related genotypes. Scores are represented with solid black squared symbols when positive and empty when negative. Square size is relative to the score value.

Tests for spatial structure indicated the existence of global spatial structures within cohorts (max(t) = 0.019 for both cohorts, p = 0.016 and 0.036 for 2010 and 2011 respectively), but no local structures, e.g., genetic differentiation among close neighbours, (max(t) = 0.020 for both cohorts, p = 0.460 and 0.126 for 2010 and 2011). The sPCA analyses showed that most of the data structure was explained by the first three principal components (PC) in 2010 and by the first two in 2011 (Figure S1.A). The eigenvalues of these PCs were 0.045, 0.030 and 0.025 in 2010, and 0.040 and 0.027 in 2011, with the remaining eigenvalues showing lower values (Figure S1.B). For environment-genotype association studies, we retained the first two PCs, as these stand out in terms of genetic variance and spatial autocorrelation (Figure S1.A). The global structure represented by the first PC (PC1) from the sPCA, revealed similar spatial genetic patterns for both cohorts, extending as a cline along the X-axis of our study plot (Fig. 2), while the second (PC2) did not revealed a clear spatial pattern (Figure S2.B).

### Isotropic spatial genetic structure

**Figure 3:**
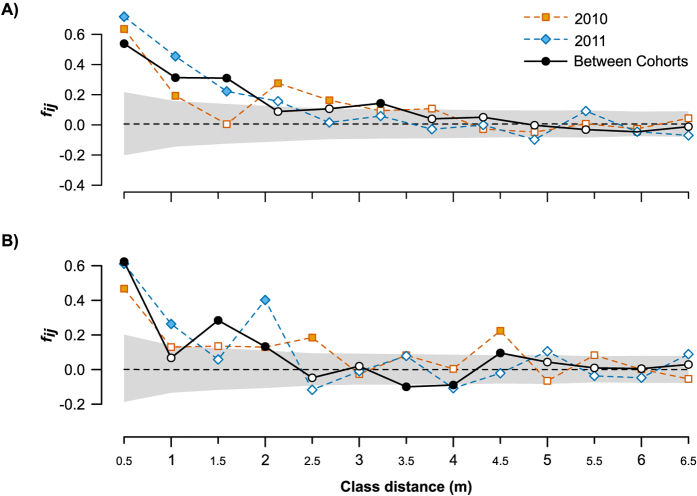
Isotropic distograms. For nuclear (**A**) and plastid (**B**) markers, the average kinship at each distance class are plotted with differing symbols depending on the comparison: orange squares (2010), blue diamonds (2011) and black circles (comparisons between cohorts). Filled symbols denote significance of the value when compared with the null hypothesis of no isolation by distance. Grey filled areas represents 95% confidence intervals for the null hypothesis of no spatial structure in the between-cohort comparisons.

Kinship-distance distograms performed using nuclear markers showed positive significant values only at short distances (<2.5 m; Fig. 3A). The significant positive values for the first distance class (0.063 ± 0.013 in 2010; 0.071 ± 0.020 in 2011) and the negative slope of the relationship between kinship and the natural logarithm of spatial distance (−0.011 for both cohorts) lead to the same significant value of intensity of SGS (*Sp = *0.012 ± 0.001; Table 2). The analysis of the plastid haplotypes showed similar results, with positive and significant values below 0.5 and 1 m in 2010 and 2011 respectively, in addition to occasional significant values at further distances (Fig. 3B). The *F*_*1*_ and *Sp* coefficients depicting SGS intensity showed significant values for both cohorts (*F*_*1*_ = 0.467 and 0.627; *Sp* = 0.207 and 0.019 in 2010 and 2011 respectively; p < 0.05).

We found similar patterns for the isotropic SGS between cohorts. When plotting the kinship coefficients of plants belonging to different cohorts against distance we observed that the pattern was maintained for both types of marker. We obtained significant positive values of average kinship coefficient below 1.5 m for the nuclear markers (Fig. 3A) and below 2 m for the plastid marker (Fig. 3B). Moreover, there were non-significant between-cohort differences in *F*_*1*_ and *Sp* (Table 2).

### Anisotropic spatial genetic structure

**Figure 4:**
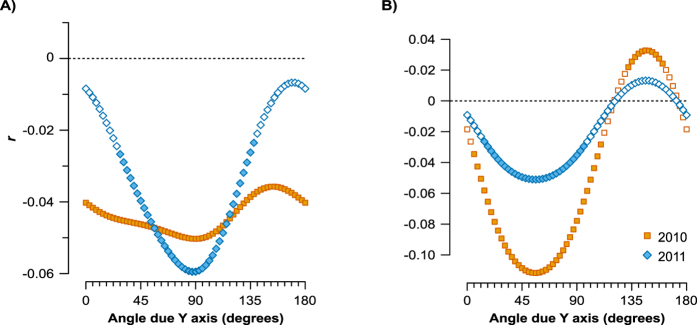
Bearing correlograms. For a series of bearing angles from 0 to 180° due the Y-axis of the plot, the Mantel correlation coefficient between genetic similarity and transformed distance matrix is plotted for both cohorts and for the nuclear (**A**) and plastidial (**B**) markers. Orange squares denote values for the cohort in 2010; blue diamonds values for the cohort in 2011. Significance after permutation is represented with filled symbols.

The correlation between kinship and spatial distance varied with compass directions for each cohort and marker, indicating the occurrence of anisotropy in SGS (Fig. 4). This variation coincided between cohorts such that there was congruence in the bearing angles with the strongest and weakest SGS for each marker. For the nuclear markers, both cohorts presented the strongest correlation along the X-axis of the study plot (*r* = −0.050, *θ*_*s*_ = 90° in 2010; *r* = −0.059, *θ*_*s*_ = 87° in 2011; Table 3; Fig. 4A), and the weakest correlation at relatively close angles (*r* = −0.036, *θ*_*w*_ = 154° in 2010; *r* = −0.007, *θ*_*w*_ = 171° in 2011). Plastid markers showed the same bearing angle with the strongest correlation (*θ*_*s*_ = 56°, *r* = −0.112 and −0.051 in 2010 and 2011; Table 3; Fig. 4B) similar to the bearing angle with the weakest correlation (*θ*_*w*_ = 146°, *r* = 0.033 and 0.013 in 2010 and 2011 respectively).

For the nuclear markers the strength of the correlation at *θ*_*s*_ showed the same values (*Sp* = 0.013 in 2010 and 2011), while at *θ*_*w*_ these were different (*Sp* = 0.020 and −0.007). The plastid marker showed differing values of strength at both angles (*θ*_*s*_: *Sp* = 0.107 and 0.032; *θ*_*w*_: *Sp* = 0.058 and −0.011).

### Environment-genotype correlations

The lagged autoregressive models performed on PC1 and PC2 presented low values of Moran’s I index in the residuals (<0.099), indicating a good performance of these models (Supplementary Table S1), while *ρ,* the parameter associated with the inherent spatial autocorrelation term, was the most important parameter with values consistently above 0.8 (Table 4). The averaged parameters from the selected models indicated some environment-genotype associations. Light availability (DSF) was strongly associated in both cohorts with PC1 (*w+ = *1 and 0.585 for 2010 and 2011; Table 4; Fig. 2) and in a lesser extent with PC2. Anions were strongly associated with PC1 in 2011 (*w+ = *0.681; Figure S2.A) and PC2 in 2010 (*w+ = *0.479), whereas cations had the strongest effect on PC2 both years (*w+ = *0.721 and 0.438 for 2010 and 2011 ; Figure S2.B).

## Discussion

We did not find genetic differentiation between two consecutive cohorts of the biennial plant *E. mediohispanicum*, a result indicating that these cohorts belong to the same gene pool and behave cohesively. Furthermore, fine scale SGS was congruent between cohorts, indicating recurrent seed dispersal limitation among years. Additionally, we found an association between the microenviroment and the spatial genetic variation. Below, we discuss the SGS found within the studied population and the causes that may be inducing it. We end by discussing how the absence of genetic differentiation and the maintenance of the SGS may be driven by inter-annual migrants between cohorts.

The spatial distribution of genotypes deviated each studied cohort from what it is expected under a random distribution of alleles, indicating the existence of SGS. Particularly, the genotypes were more similar at short distances, fitting a pattern of solation by distance even at this fine spatial scale. These results are supported by the global structure found using the sPCA and by the Mantel correlograms for both plastid and nuclear markers. This pattern emerges from restricted gene flow driven by the local dispersal of seeds or pollen, whose contributions to the final SGS may differ^31^. Limited seed dispersal promotes siblings to germinate and establish locally, creating aggregations of relatives and therefore structuring genotypes at the fine-scale^6^,^7^. This is likely to have happened in our study population because the distances at which the significant positive correlation vanished (<1–2.5 meters) are consistent with the reported distances of seed dispersal in *E. mediohispanicum* (0–0.38 m)^29^. However, limited seed dispersal may not be acting alone. The congruent SGS pattern showed by the nuclear and plastidial markers suggest that pollinators moving pollen at long distances are not blurring the SGS. In addition, we think that the spatial aggregation of individuals (Figure S3) together with the prevalence of floral visitors that tend to forage at short distances^32^,^33^, may facilitate mating events among nearby relatives, causing biparental inbreeding, and reinforcing the observed SGS. Nevertheless, even under random mating, SGS can occur as a byproduct of limited seed dispersal^8^,^15^, and therefore further analyses are needed to disentangle the relative role of pollen and seeds dispersal as drivers of the observed pattern.

We found asymmetry in SGS for both cohorts, indicating directionally biased gene flow. Kinship-distance correlation varied periodically along the spatial directions, revealing higher resistances to gene flow at 90 and 87° due the Y-axis of the plot (Fig. 4). Directionally biased gene flow has been related to spatial gradients on environmental factors^13^, and has been reported in plant species where topography or environmental factors reinforce SGS in particular directions^10^,^11^,^13^,^34^. If these gradients are maintained over years, the predominance of gene flow along some axes may leave their signature in the SGS. However, we did not identify any environmental gradients because the analysed environmental factors showed a marked patchy heterogeneity. Several factors acting locally could produce the observed SGS asymmetry. For example, the non-random movement of pollinators driven by preferences for plant traits, or micro-environmental factors, may produce heterogeneous patterns of gene flow^12^. These environmental factors could also affect seed germination and seedling establishment, contributing to directionally biased SGS^35^. Additionally, directional genetic flow from other populations could contribute to the observed pattern, but at the present we have no evidences for this to be considered as an important factor shaping SGS asymmetry.

Site-specific ecological conditions mediate genetic connectivity^36^. In our study, the spatial variation in allele frequencies showed by the PC1 matched the spatial variation in understory light availability (Fig. 2). This pattern could be produced by light modulating the suitability of spaces for seed germination and seedling establishment^37^, and therefore contributing to a kind of fine-scale isolation-by-environment^38^. In addition, this pattern could be reinforced by the influence of light on the identity and behaviour of the pollinators visiting the plants^39^. Under this scenario, plants sharing the same light conditions would be visited by similar pollinators and therefore may exhibit higher genetic connectivity. This hypothesis, however, needs to be tested by further analyses of paternity on the offspring to relate pollen flow with understory light conditions and pollinators.

We also found that SGS was associated with some soil variables. Effects of soil heterogeneity on the pattern of genetic variation have been reported in several plant species. For example, Segarra-Moragues *et al.*^35^ have recently shown significant genetic differentiation between rosemary plants (*Rosmarinus officinalis*) growing in siliceous *vs.* calcareous soils. In our case, the observed fine-scale soil-SGS association could be caused by the differential performance (establishment, survival, growth) of some genotypes in slightly different edaphic conditions. However, it is noteworthy to highlight the high values of *ρ* (>0.867) in our models, indicating that, at this scale, spatially limited dispersion prevails over these site-specific environmental factors. In this sense, more evidence is necessary to disentangle the importance of the genotype-environment interactions in shaping SGS at this fine scale.

Prudence is cautioned in interpreting spatially lagged regressions because little is known about their performance when applied to landscape genetic analyses^40^. However, we are confident of our results for a number of reasons. It has been shown that these models are robust to type I errors^41^,^42^ and do not fail when using sPCA principal components as response variables^40^,^43^. In addition, *ρ* was consistently high in our models, and the residuals showed little to no spatial autocorrelation (Table S1), indicating that these models accounted adequately for the spatial autocorrelation. Nevertheless we are aware that the associations found here have to be interpreted cautiously when inferring the causal mechanisms leading to these environment-genotype associations.

Plants flowering in different years did not differ in their allelic frequencies, indicating that the two studied cohorts belong to the same gene pool. This is confirmed by the low values of *F*_*ST*_ and the non-significance of the AMOVA. In small populations of biennial plants, such as *E. mediohispanicum*, recurring and catastrophic declines of population size could conduce to genetic differentiation even among cohorts, since these fluctuations in population size are expected to produce intense genetic drift in small populations^26^. But even in the case of large demographic events, the occurrence of gene flow between cohorts may counterbalance genetic differentiation. Gene flow is surely due to the high amount of inter-annual migrants, up to 17% according to our Bayesass estimates, a high value when compared to most studies using this same algorithm^44^. Several factors may promote between-cohort gene flow via different pathways in *E. mediohispanicum* (Figure S4). Seed dormancy can be activated by unfavourable weather conditions^45^ and leads to mating between the descendants of different cohorts^25^. In addition, the arrival of inter-annual migrants can take other different paths. Firstly, via the repeated flowering of some individuals in the oncoming year. This could be caused by delayed reproduction or via a reduced reproductive success during a year followed by subsequent resource allocation, permitting a new reproductive event in the following year^46^. In our study species, several factors such as herbivory^47^ or pollen limitation^48^ may trigger the existence of this type of inter-annual migrants. Additionally, delayed or precocious flowering can occur as a response to climatic suitability to plant growth^46^, a likely candidate factor considering the climatic unpredictability of Mediterranean ecosystems^49^. Whatever the reasons, our results demonstrate that inter-annual migrants prevent the genetic differentiation among cohorts, contributing to population cohesion.

Between-cohort gene flow, besides homogenizing allelic frequencies, also reinforced SGS. Firstly, kinship-distance correlations showed a similar pattern within and between cohorts, with the same significant positive values spanning up to 1.5 and 2 m for nuclear and plastid markers respectively. Along with this, SGS intensity did not differ significantly between cohorts, emphasizing the similarities in isotropic SGS. These findings confirm that plants growing nearby are genetically more similar independently of whether they belong to the same flowering cohort or not. In addition, SGSs were similar in their spatial asymmetry, with coinciding bearing angles for the strongest and weakest correlations. The maintenance of the SGS between cohorts is likely due to the limited dispersal of seeds. The integration of all individual seed shadows determines the starting template for the spatial genetic patterns of future flowering plants^16^,^50^. Therefore, an inter-annual migrant stands as a representative of this spatial template in the new cohort. From this localized migration spot, the oncoming processes of gene flow through pollination and seed dispersal will spread the migrant genes in the new cohort similarly to what would occur in its parental cohort. In this sense, the environmental factors will similarly favour or restrict gene flow in some directions, and therefore migrant alleles will flow asymmetrically. These recurrent migration events will, in the long term, match the spatial genetic variation among cohorts, leading to the observed congruence in SGS. Furthermore, between-populations migration could also contribute to produce the observed SGS. However, biased and recurrent migration, probably mediated by long-distance pollination events, would be needed to explain the observed pattern. As these pollination events do not contribute to the plastidial SGS, the importance of recurrent spatial migration in the temporal maintenance of the SGS is, at least, doubtful.

We have demonstrated the existence of congruent fine-scale spatial genetic variation in two consecutive cohorts of a natural population of the biennial plant *E. mediohispanicum*. This SGS was consistently associated with environmental factors, such as light availability and soil composition. More importantly, there was always a strong spatial autocorrelation in SGS, suggesting that SGS was mostly caused by the spatial pattern of limited seed dispersal in distance rather than by any environment-genotype association. The two studied cohorts had homogenized allelic frequencies, indicating the absence of genetic isolation and the existence of inter-annual migrants. These migrants, due to spatial limited seed dispersal exhibited by *E. mediohispanicum*, imprint a spatially localized genetic signature on the new cohort, which ultimately results in a between-cohort matching of SGS.

## Materials and Methods

### Sampling design

In years 2010 and 2011 we set up a 20 × 20 m plot delimiting a population of *E. mediohispanicum* situated at 1723 m.a.s.l. at the Sierra Nevada Protected Area (SE Spain; 37° 8.07′ N 3° 21.71′ W). Each year we randomly selected 100 flowering plants and mapped their spatial location at the centimetre level (Fig. 1). These plants represented ~ 85% of the total individuals comprising a reproductive cohort. We characterized light and soil conditions for each individual plant. Light availability, measured as Direct Site Factor (DSF), was derived from hemispherical photographs taken at the ground level using a Nikon coolpix 4500 and subsequently analysed by applying the Hemiview software (Delta-T Devices, Cambridge, UK). Soil was characterized by means of cations (Na+, K^+^ and Mg^2+^) and anions (total nitrogen and phosphorous), and moisture content at field capacity as a reliable measure of the soil’s ability to retain water (see Supplementary Information S1). To decrease variable dimensionality we ran a PCA over cations and anions independently. We found two components explaining cation variability: the first (cations 1) explaining 58% and was associated to Mg^2+^ and K^+^ and the second (cations 2) explaining 33% and was associated to Na^+^ (Figure S5). Anions were synthesised in a variable explaining 70% of the variability and equally associated to nitrogen and phosphorous.

### Genetic characterization

We genotyped each plant using nuclear and plastid molecular markers. We isolated DNA using the GenElute Plant Genomic DNA Miniprep Kit (Sigma-Aldrich) on 60 mg of plant material previously crunched in liquid nitrogen.

We amplified the plastidial *trnL-trnF* spacer (~1300 bp) using the tabC and tabF primers (see Abdelaziz *et al.*^51^ and Supplementary Information S2). PCR products were mixed with 0.15 volumes of 3 M sodium acetate, pH 4.6, and 3 volumes 95% (v/v) ethanol and subsequently precipitated after centrifuging at 4 °C. Amplicons were then sent to Macrogen (Geumchun-gu, Seoul, Korea) for sequencing in both directions, using the respective PCR primers. Chromatograms were reviewed and contigs were produced using Geneious v.7^52^ (Biomatters, http://www.geneious.com/). Sequences were uploaded to Genbank (accession numbers KX641272 to KX641276).

From each plant we amplified 10 unlinked nuclear microsatellites loci (SSR) described in Muñoz-Pajares *et al.*^53^ (Supplementary Information S2). We additionally genotyped 500 offspring in order to detect and correct genotyping errors (e.g., allele dropout or null alleles) in the parental plant genotypes. Electropherograms were analysed and genotypes called using PeakScanner v.2 (Applied Biosystems) and exhaustive eye-inspection. We characterized each microsatellite locus within cohorts by computing observed and expected heterozygosity, number of alleles, and inbreeding coefficient. For this later we tested if deviated significantly from zero using 999 bootstrap replicates. We tested for Hardy-Weinberg equilibrium using an exact test based on Monte Carlo permutations for each locus and for all loci together. These tests were performed using the ‘pegas’^54^ and ‘diveRsity’^55^ packages in the open source software R v. 3.2.2.

### Inter-annual genetic structure

We evaluated the amount of genetic differentiation between cohorts using Wright’s F_ST_ statistic. The significance of this statistic was obtained by comparing with a null distribution obtained after 500 random permutations of genotypes. We also performed an AMOVA between cohorts using Chord’s distances among genotypes^56^. Moreover, we used Bayesass v.1.3^57^ to infer rates of recent inter-annual migrants. This software uses Markov chain Monte Carlo techniques to estimate the posterior probabilities of recent individual immigrants. We ran three independent MCMC runs for 10^7^ iterations with a thinning of 2000 and a burn-in of the first 10% of the iterations. Delta parameters of allele frequency, migration, and inbreeding were set as 0.15.

### Spatial variation of allele frequencies

We checked for the occurrence of fine-scale SGS within each cohort by conducting a spatial principal component analysis (sPCA)^58^ on the nuclear markers. This multivariate ordination technique tests for the existence of global structures (such as spatial clines) or local structures (genetic differences between neighbours). sPCA integrates principal component analysis and Moran’s autocorrelation index to reduce the multidimensional nature of genotype data into a set of highly informative orthogonal vectors that differentiate spatial patterns of genetic variation. To accomplish this analysis, a connection network depicting spatial weights among individuals has to be previously defined. We used the inverse of the spatial distances among pairs so that all plants were considered neighbours while accounting for the spatial cost distance. With the resulting sPCA principal components, we assessed spatial structures by representing the scores of the two main vectors in space. We only used the two first components (PC1 and PC2) to reduce the burden of the computation to the most informative sPCA principal components. We evaluated the significance of global and local structures using the test proposed by Jombart *et al.*^58^.

### Isotropic spatial genetic structure

We explored the extent and intensity of SGS to evaluate if relatives were spatially clumped as a result of isolation by distance. For each cohort and for each nuclear (SSR) and plastid marker, we calculated Nason’s kinship coefficients^59^, which are standardized to a population’s allele frequencies and are highly robust to HWE deviations^1^. When calculating this coefficient using the plastid marker, we treated haplotypes as alleles from a haploid organism. To explore the spatial extent of SGS we calculated the average kinship coefficient for a set of distance classes (*F*_*D*_) spanning 0.5 meters, and plotted them against distance^60^. We subsequently measured the intensity of SGS by calculating two gene dispersal parameters: *F*_*1*_, the average kinship coefficient for the first distance class (<0.5 meters), and the *Sp* coefficient^1^, defined as -*b*_*F*_/(1-*F*_*1*_), where *b*_*F*_ is the slope of the regression of kinship coefficients on the natural logarithm of the spatial distance among individuals within an optimized range of distances. The range of distances for which *b*_*F*_ is calculated is related to the average parent-offspring distance and its approximation follows an iterative procedure^1^ implemented in the software SPAGeDi 1.5a^61^. Standard errors for all parameters were calculated jackknifing over SSR loci^1^,^9^, except in the case of cpDNA haplotypes, where only a marker was available. For SSR *Sp*, and each SSR *F*_*D*_ we obtained a null distribution of expected values under the hypothesis of no SGS by permuting genotypes among individuals 9999 times, and compared them with the observed values to obtain a significance value.

We investigated the maintenance of the isotropic SGS between cohorts by plotting kinship coefficients against spatial distance as explained before, but restricting the comparisons to individuals belonging to different cohorts. Then, we compared the intensity of the SGS between cohorts by a t-test using the average and standard errors obtained by jackknifing over loci. We also calculated *b*_*F*_, *F*_*1*_ and *Sp* dispersal parameters restricting the comparisons to individuals belonging to different cohorts.

### Anisotropic spatial genetic structure

We explored the anisotropy of SGS by testing whether its strength varied in different spatial directions using Rosenberg’s^62^ bearing correlogram, an analysis that uses a Mantel test to correlate a genetic similarity matrix with a transformed distance matrix for a set of spatial directions, measured as the angles formed with the Y-axis (*θ*). For a given direction, the transformed distance matrix is obtained by first calculating the natural logarithm of the original distance matrix, followed by weighting its values by the squared cosine of the clockwise bearing angle depicted by each pair of individuals and the fixed spatial direction. For this analysis we used the obtained kinship matrix and a set of 128 equidistant bearing angles. The significance of the correlations was obtained using a permutation test (999 permutations). Through this analysis we were able to find the bearing angle denoting the strongest (*θ*_*s*_, minimal Mantel correlation) and weakest SGS directions (*θ*_*w*_, maximal Mantel correlation). Next, we calculated the strength of SGS at *θ*_*s*_ and *θ*_*w*_ for each cohort. To perform this, and to augment the number of paired kinship coefficients, we included the plants inside a range of 30° around *θ*_*s*_ and *θ*_*w*_^10^. For each cohort and *θ*_*s*_ and *θ*_*w,*_ we computed *Sp* and *F*_*1*_ and obtained their significance as explained before. These analyses were performed using SPAGeDi 1.5a^61^ and personalized scripts in R (Supplementary Information S3). As the temporal maintenance of anisotropy is determined by the congruence and strength of *θ*_*s*_ and *θ*_*w*_ between cohorts, we therefore compared the gene dispersal parameters *Sp* and *F*_*1*_ by means of a t-test.

### Environment-genotype correlations

We evaluated the contribution of environmental factors to the spatial genotypic variation beyond isolation by distance. We used lagged simultaneous autoregressive models using the first two components of the sPCA as response variables^43^. These models assume the inherent spatial autocorrelation occurring in the response variable and are fairly robust to type I errors^41^,^42^. The models take the form:


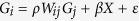


In this equation, the typical ordinary least squares regression (*G*_*i*_* = βX + ε*) is modified by adding a term (*ρW*_*ij*_*G*_*j*_) which controls for the inherent spatial autocorrelation which is assumed to occur in the response variable. The PC scores of all other individuals (*G*_*j*_) are weighted by the parameter *ρ*, which accounts for the lack of independence among individuals, and by the cost-distance-weighting matrix (*W*_*ij*_). We computed *W*_*ij*_ using the inverse spatial distances among individuals because it approximately emulates a spatial autocorrelation under IBD^40^,^43^. By using site-based measures we were able to determine the environmental factors associated with genetic structure^63^.

From all possible combinations of models we selected those with ΔAIC < 2 from the best model. Following, we model-averaged parameter estimates and calculated their relative importance following Burnham and Anderson^64^. Moreover, the selected models were checked for remaining autocorrelation in residuals by using a Moran’s I test. Model performances and selection were calculated using the R packages ‘spdep’^65^ and ‘AICcmodavg’^66^.

## Additional Information

**How to cite this article**: Valverde, J. *et al.* Inter-annual maintenance of the fine-scale genetic structure in a biennial plant. *Sci. Rep.* **6**, 37712; doi: 10.1038/srep37712 (2016).

**Publisher's note:** Springer Nature remains neutral with regard to jurisdictional claims in published maps and institutional affiliations.

## Supplementary Material

Supplementary Information

## Figures and Tables

**Table 1:** Population genetic parameters per locus and year (cohort).

Locus	2010						2011					
	R_S_	% MD	F_IS_	H_O_	H_O_/H_E_	χ^2^	R_S_	% MD	F_IS_	H_O_	H_O_/H_E_	χ^2^
C5	10	0	−0.024	0.756	1.024	21.670	10	0	0.043	0.766	0.957	25.373
D4	8	0	0.027	0.558	0.973	11.625	8	0	−0.025	0.588	1.025	45.185
E4	14	1	0.053	0.794	0.946	148.649	15	0	0.064	0.816	0.936	**92.432**
D2	22	0	0.053	0.912	0.947	200.625	21	0	**0.076**	0.925	0.924	211.690
E3	4	0	0.094	0.244	0.906	7.963	6	3	0.062	0.287	0.938	14.710
E6	13	0	−0.057	0.694	1.057	47.827	12	0	0.030	0.684	0.969	40.239
E8	15	0	**0.192**	0.758	0.808	**204.151**	16	0	**0.170**	0.751	0.830	166.314
E5	16	0	0.052	0.869	0.948	117.717	14	1	**0.123**	0.857	0.877	**143.413**
D10	5	0	−0.022	0.531	1.022	5.928	5	1	−0.043	0.506	1.043	13.180
D11	6	0	−0.043	0.520	1.043	9.327	5	1	0.079	0.529	0.921	7.506

For each locus and cohort: allelic richness (R_S_), percentage of missing data (%MD), inbreeding coefficient (F_IS_), observed heterozygosity (H_O_), departure from the expected heterozygosity under Hardy-Weinberg equilibrium (H_O_/H_E_), and their corresponding chi-squared values are shown. Significant values are indicated in bold.

**Table 2:** Isotropic SGS strength within and between cohorts.

Marker	Cohort	*b*_*F*_	*F*_*1*_	*Sp*
SSR	2010	**−0.011 ± 0.001**	**0.064 ± 0.013**	**0.012 ± 0.001**
	2011	**−0.011 ± 0.001**	**0.072 ± 0.020**	**0.012 ± 0.001**
	Between	**−0.011 ± 0.001**	**0.063 ± 0.016**	**0.012 ± 0.001**
t-test between cohorts		1	0.751	1
cpDNA	2010	**−0.110**	**0.467**	**0.207**
	2011	**−0.007**	**0.627**	**0.019**
	Between	**−0.083**	**0.624**	**0.220**

*b*_*F*_: kinship-log spatial distance regression slope within an optimized range of distances. *F*_*1*_: average kinship for the first distance class. *Sp*: slope of the regression of kinship on the logarithm of the spatial distance, it equals to –*b*_*F*_/[1 – F_1_]. Between cohorts parameters were obtained restricting the comparisons to individuals belonging to different cohorts. Significant values are indicated in bold. P-values of the t-tests comparing between cohorts are also shown.

**Table 3:** SGS strength within cohorts at the bearing angles with the strongest and weakest kinship-distance correlations.

Marker	Cohort	*θ*_*s*_				*θ*_*w*_			
		*θ*	*b*_*F*_	*F*_*1*_	*Sp*	*θ*	*b*_*F*_	*F*_*1*_	*Sp*
SSR	2010	90°	**−0.013 ± 0.003**	−0.001 ± 0.001	**0.013 ± 0.003**	154° 42′	**−0.020 ± 0.006**	0.000 ± 0.001	**0.020 ± 0.006**
	2011	87° 11′	**−0.013 ± 0.005**	0.000	**0.013 ± 0.005**	171° 34′	0.007 ± 0.003	−0.001 ± 0.001	−0.007 ± 0.003
t-test between cohorts			0.962	0.782	0.962		0.000	0.426	0.000
cpDNA	2010	56° 15′	**−0.104**	**0.029**	**0.107**	146° 15′	−0.059	**−0.020**	0.058
	2011	56° 15′	−0.032	0.005	0.032	146° 15′	0.011	**−0.011**	−0.011

For the compass directions with the strongest (*θ*_*S*_) and weakest (*θ*_*W*_) kinship-distance correlations, we show their bearing angles (*θ*) and SGS strength parameters *b*_*F*_, *F*_*1*_ and *Sp*. P-values of the t-tests comparing between cohorts are also shown.

**Table 4:** Model-averaged parameter estimates after model selection.

		2010			2011		
		Estimate	SE	*w+*	Estimate	SE	*w+*
PC1	*ρ*	0.883	0.078	1	0.874	0.087	1
	Light availability (DSF)	0.158	0.041	1	0.036	0.042	0.585
	Anions (N and P)	−0.004	0.019	0.135	−0.060	0.059	0.681
	Cations 1 (Mg^2+^ and K^+^)	−0.049	0.050	0.632	0.046	0.063	0.498
	Cations 2 (Na^+^)	0.003	0.013	0.140	−0.001	0.014	0.078
	Field capacity	−0.054	0.042	0.807	−0.001	0.021	0.079
PC2	*ρ*	0.899	0.068	1	0.927	0.051	1
	Light availability (DSF)	−0.035	0.040	0.596	−0.004	0.018	0.217
	Anions (N and P)	−0.034	0.045	0.479	−0.001	0.021	0.158
	Cations 1 (Mg^2+^ and K^+^)	−0.053	0.049	0.721	−0.022	0.041	0.438
	Cations 2 (Na^+^)	−0.125	0.028	1	−0.008	0.026	0.244
	Field capacity	0.008	0.020	0.268	0.014	0.038	0.326

Regression model parameters with estimates, standard errors, and relative importance values (*w*+) resulting from the model selection. ρ denotes the parameter associated with the inherent spatial autocorrelation term.

The *w*+ values of *ρ* equals to 1 because this is a structural parameter of the lagged simultaneous autoregressive models.
