# Supplementary material for: Inter-annual maintenance of the fine-scale genetic structure in a biennial plant
Source: Sci Rep. 2016 Nov 24;6:37712. doi: 10.1038/srep37712 (PMC5121606; doi:10.1038/srep37712)

Supplementary Information

Inter-annual maintenance of the fine-scale genetic structure in a biennial plant

Javier Valverde[1], José María Gómez[1,2], Cristina García[3], Timothy F. Sharbel[4], Mª Noelia Jimenez[5], Francisco Perfectti[6]

[1] Dpto de Ecología, Universidad de Granada, E-18071 Granada, Spain

[2] Dpto de Ecología Funcional y Evolutiva, Estación Experimental de Zonas Áridas (EEZA-CSIC), E-04120 Almería, Spain

[3] Plant Biology, CIBIO/InBio, Centro de Investigação em Biodiversidade e Recursos Genéticos, Laboratório Associado, Universidade do Porto. Campus Agrário de Vairão, 4485-661 Vairão, Portugal

[4] Leibniz Institute for Plant Genetics and Crop Plant Research (IPK), 06466 Gatersleben, Germany. Present address: Global Institute for Food Security (GIFS), University of Saskatchewan, Saskatoon, Canada

[5] Dpto de Botánica, Universidad de Granada, E-18071 Granada, Spain

[6] Dpto de Genética, Universidad de Granada, E-18071 Granada, Spain

Supplementary Methods S1. Soil analyses

During 2010 we collected soil samples under each individual plant to a depth of 0-10 cm. We measured moisture content at field capacity extracted in a pressure plate at -33 kPa according to Cassel and Nielsen[1]. We estimated the amount of exchangeable cations (Na, K^+, Ca^{2+}, Mg^{2+}) by extracting with NH4OAc 1N. Ca^{2+} and Mg^{2+} were measured by atomic-absorption spectroscopy and Na and K^+ by flame photometry. Finally, total nitrogen was inferred by the Kjeldahl method[2], and available P using the classical Olsen method[3]. Soil variables for plant in 2011 were estimated using kriging interpolation. Before PCA analysis, all variables were log-transformed in order to normalize and afterwards centered and scaled by their standard deviations.

Supplementary Methods S2. DNA amplifications

trnL-trn-F

PCR reactions for the chloroplastidial trnL-trnF intergenic spacer was performed using the tabC and tabF primers[4]. Following Abdelaziz et al.[5], we performed these amplifications in 25 µL reaction volume, including 0.5 units of Taq DNA polymerase, 2,5 µL 10x reaction buffer containing MgCl 2 at 1.5 mM, 0.2 mM each dNTP (all product from New England BioLabs), 0.2 µM each primer, and 5 ng of template DNA. Amplification conditions included 35 cycles with 94 °C (15 s) denaturation, 58 °C (30 s) annealing, 72 °C (90 s) extension, and 72 °C (3 min) final extension.

Microsatellites

The PCR reactions were performed in 14 µL reactions including 0.3 units of Taq polymerase (New England BioLabs), 1.4 µL 10× buffer containing MgCl2 at 1.5 mM (New England BioLabs), 0.16 mM of each dNTP, 0.33 µM for each primer and 2.5 ng of DNA template. We used a Gradient Master Cycler Pro S (Eppendorf) to perform PCRs. The initial denaturing step consisted in 3 min at 94 °C, followed by 35 to 40 extensión cycles consisting in 15 s at 94 °C, 30 s at a specific anealing

temperature (see table below) and 90 s at 72 °C, with a final extension step of 3 min at 72 °C.

Table Characteristics of the 10 microsatellite markers. Adapted from Muñoz-Pajares et al.[6].

Locus	Primer sequence (5'- 3')	Repeat motif	Product size (bp)	T_a (ºC)
C5 (JF766210)	F: TCTTTCTTCTGCGGTTTATTC<br>R: CGTTTTTGTTGTTGTTCTGG	CCA_8	164 – 182	56
D2 (JF766211)	F: ACGGAAGATGACGATGATCGACTG<br>R: CAATGTCCCTAATTGGTCAATGG	CAT_{23}	117 – 189	54
D4 (JF766212)	F: TAAGGTGTTACCGGATTGTC<br>R: GTGACGATTCGCTCCTTG	ATC_7	200 – 215	57
E4 (JF766213)	F: CCTTCCTCCGACTACTCTCC<br>R: TGAGCGACTGATGATGATTC	CT_{20}	145 – 178	57
E8 (JF766214)	F: AGCTCACAGCCGTCGATGTTTGC<br>R: GAGGTGAAATACACGTAGAACCT	CT_{50}	157 – 229	50
D11 (JF766215)	F: TCCAGGGTCTGAGTCAATATG<br>R: TTACCACTCCTTGCTTCTGAA	TCA_{14}	179 – 197	53
E6 (JF766216)	F: CTTGTAACCGAGCCACTCA<br>R: ATACGGAGAAGAAAGCGAATC	TC_{14}	131 – 159	53
D10 (JF766217)	F: ACTGCCATCAAACGACCTC<br>R: TTGGTTGGAAAAGGGATTG	TCA_{12}	166 – 185	53
E5 (JF766218)	F: TCCATTTACACAATCCGTTCAT<br>R: CCAACCTGACATCTTTGCTTC	GA_{13}	167 – 195	50
E3 (JF766219)	F: TTCCTCCAGATGAAACTACACAGG<br>R: ACTTACATCGGATCGGTTGAG	GA_{17}	215 – 253	56

For each locus the GenBank accession number is given in parenthesis, forward (F) and reverse (R) primers, repeat motif, allele size ranges, and optimal annealing temperatures (T_a) are indicated.

Supplementary Methods S3. R code for bearing correlograms

The following script performs a bearing correlogram, an analytical tool for determining the directions of maximum and minimum spatial-genetic correlation[7]. This method weights the pairwise distance matrix for a set of directional angles. For a given directional angle (θ) the log-transformed distance between two points ($log\ r_{ij}$) is weighted by the squared cosine of the angle formed between their associated vector and the directional angle of study ($cos^2\ (\theta-\alpha_{ij})$).

$$d_{ij}= log\ r_{ij}\ cos^2\ (\theta-\alpha_{ij})$$

The correlation between the kinship and the resulting weighted distances is then tested via Mantel test, and the statistical significance obtained by through a permutational procedure. The script uses functions included in the packages 'geosphere'[8], 'aspace'[9], 'RandomFields'[10] and 'ecodist'[11].

Usage:

bearing.analysis (K, coord, divisions = 64, perms=1000)

Arguments:

K	a numeric matrix containing all paired kinship values
coord	data frame with each individual coordinates
divisions	a numeric variable denoting the number of angle divisions between the 0 and the 180 degrees from the (0,1) directional
perms	a numeric variable defining the number of permutations used to obtain the statistical significance of the Mantel test

Code:

```
bearing.analysis <- function(Kij, coord, divisions=64, perms=1000)
{
	Kij <- as.matrix(Kij)

	# required libraries
	require(geosphere)
	require(aspace)
	require(ecodist)

	# Obtain the logarithm of the distance matrix
	log.Rij <- log(dist(coord))

	# set divisions in radians
	angle <- seq(0,360,360/divisions)
	angle <- angle[-length(angle)]

	# data frame to store for each angle mantel test data:
	main <- data.frame("angle"=angle, "radians"=NA, "r.Mantel"=NA, "p.val1"=NA, "p.val2"=NA, "p.val3"=NA,"color"=NA)

	# set angles to radians
	angle <- as_radians(angle)

	# set matrix to store the bearing angles for each pair of points
	alphas <- Kij; alphas[] <- NA

	# Loop for setting bearing angles in the M matrix
	for(i in 1:nrow(coord))
	{
		for(j in 1:nrow(coord))
		{
```

```
                    # this returns angles in degrees
            alphas[i,j]<-bearingRhumb(coord[i,],coord[j,])
            }
    }

    # transform bearing degrees to radians.
    alphas <- as_radians(alphas)

    # Loop for each angle
    for (t in 1:length(angle))
    {
            print(paste("angle:",t))

            # Theta. Reference angle (in radians)
            main[t,"radians"] <- theta <- angle[t]

            # Transformed distance matrix
            cos2.ang <- cos(theta-alphas)^2
            Dij <- as.matrix(log.Rij)*cos2.ang

            # Mantel test using the kinship matrix (Kij) and the weighted spatial distance matrix (Rij).
            # Permutations (user defined in 'perms' argument) are<used for significance.
            main[t,3:6] <- mantel(as.dist(Kij)~ as.dist(Dij), nperm = perms)[1:4]

            # Define colours for significance levels
            if(any(main[t,4:6] <= 0.05)) {main[t,7] <- "black"}
            else {main[t,7] <- "white"}

            print(paste("Mantel r:",round(m[1],4),"p-values:",m[2],m[3],m[4]))
    }

    # Plot bearing correlogram
    if(max(main$r.Mantel)<0){m <- 0}
    else {m ← max(main$r.Mantel)}
    plot(main[,1],main[,3], axes=F,type="n", ylim=c(min(main$r.Mantel),m),  ylab="Mantel r",  xlab="angle due X-axis (degrees)")
    lines(main$angle, main$r.Mantel, lty=3, col="gray50")
    points(main[,1], main[,3], pch=22, col="black", bg=main$color, cex=.6)
    axis(1, labels=seq(0,max(main$angle),22.5), at=seq(0,max(main$angle),22.5), cex.axis=.6)
    axis(2, cex.axis=.6)
    abline(h=0, lty=3, col="gray50")

    return(main)
}
```

SUPPLEMENTARY REFERENCES:

1. Cassel, D. K. & Nielsen, D. R. in *Methods of soil analysis. Part. 1: physical and mineralogical methods* (ed. Klute, A.) 901–926 (ASA-SSSA, 1986).

2. Bremner, J. M. in *Methods of soil analysis. Part. 2. Chemical and microbiological properties* (eds. Black, C. A., Evans, D. D., Esminger, L. E. & Clark, F. E.) 1324–1345 (American Society of Agronomy, 1965).

3. Olsen, S. R. & Sommers, L. E. in *Methods of Soil Analysis. Chemical and Microbiological Properties* (ed. Page, D. L.) 403–430 (American Society of Agronomy and Soil Science Society of America, 1982).

4. Taberlet, P., Gielly, L., Pautou, G. & Bouvet, J. Universal primers for amplification of three non-coding regions of chloroplast DNA. *Plant Mol. Biol.* **17,** 1105–1109 (1991).

5. Abdelaziz, M. *et al.* Using complementary techniques to distinguish cryptic species: a new *Erysimum* (Brassicaceae) species from North Africa. *Am. J. Bot.* **98,** 1049–60 (2011).

6. Muñoz-Pajares, a J. *et al.* Characterization of microsatellite loci in *Erysimum mediohispanicum* (Brassicaceae) and cross-amplification in related species. *Am. J. Bot.* **98,** e287–9 (2011).

7. Rosenberg, M. S. The bearing correlogram: a new method of analyzing directional spatial autocorrelation. *Geogr. Anal.* **32,** 267–278 (2000).

8. Hijmans, R. J. *geosphere: Spherical Trigonometry. R package version 1.4-3.* (2015). at <http://cran.r-project.org/package=geosphere>

9. Bui, R., Buliung, R. N. & Remmel, T. K. *aspace: A collection of functions for estimating centrographic statistics and computational geometries for spatial point patterns. R package version 3.2.* (2012). at <http://cran.r-project.org/package=aspace>

10. Schlather, M., Malinowski, A., Menck, P. J., Oestin, M. & Strokorb, K. Analysis, simulation and prediction of multivariate random fields with package randomfields. *J. Stat. Softw.* **63,** 1–25 (2015).

11. Goslee, S. C. & Urban, D. L. The ecodist package for dissimilarity-based analysis of ecological data. *J. Stat. Softw.* **22,** 1–19 (2007).

Table S1 Model selection for spatially lagged autoregressive models for sPC1 and sPC2. Best-ranked models are listed in ascendant AIC showing parameter estimates and values of Moran's I test over the residuals.

PC1	Light availability	Anions (N, P)	Cations 1 (Mg^{2+}, K^+)	Cations 2 (Na^+)	Field capacity	ρ	Moran's I	AICc	LogLikelihood	w_i
2010	**0.165**		**-0.075**		**-0.075**	**0.888**	0.025	91.27	-40.316	0.357
	**0.169**		-0.074	0.019	**-0.074**	**0.883**	0.023	93.144	-40.12	0.14
	**0.144**					**0.88**	**0.03**	92.509	-43.129	0.192
	**0.143**				-0.042	**0.875**	**0.027**	92.693	-42.136	0.175
	**0.173**	-0.026	-0.089		**-0.07**	**0.886**	**0.025**	93.222	-40.159	0.135
2011	0.069	**-0.1**				**0.869**	**0.026**	86.77	-39.175	0.232
			**0.117**			**0.878**	**0.032**	86.996	-40.373	0.207
	0.038		**0.101**			**0.866**	**0.028**	88.219	-39.899	0.112
		**-0.099**				**0.905**	**0.044**	88.202	-40.976	0.113
		-0.044	0.082			**0.879**	**0.031**	88.56	-40.07	0.095
	0.059	-0.079	0.032			**0.861**	**0.024**	88.788	-39.075	0.084
	0.068	-0.089			-0.019	**0.865**	**0.025**	88.922	-39.142	0.079
	0.068	**-0.102**		-0.009		**0.868**	**0.026**	88.951	-39.156	0.078

PC2	Light availability	Anions (N, P)	Cations 1 (Mg^{2+}, K^+)	Cations 2 (Na^+)	Field capacity	ρ	Moran's I	AICc	LogLikelihood	w_i
2010		-0.072	**-0.098**	**-0.127**		**0.898**	**0.045**	70.497	-29.93	0.2
	**-0.07**			**-0.125**		**0.904**	**0.06**	70.81	-31.195	0.171
	-0.057		-0.041	**-0.125**		**0.893**	**0.052**	71.235	-30.298	0.138
	-0.04	-0.057	-0.079	**-0.131**		**0.893**	**0.047**	71.541	-29.319	0.119
		**-0.083**	**-0.089**	**-0.127**	0.03	**0.901**	**0.049**	71.888	-29.492	0.1
	**-0.069**			**-0.123**	0.03	**0.903**	**0.061**	71.735	-30.548	0.108
			-0.055	**-0.117**		**0.901**	**0.052**	71.803	-31.691	0.104
	-0.041	-0.068	-0.069	**-0.13**	0.031	**0.896**	**0.052**	72.915	-28.849	0.06
2011			-0.049			**0.927**	**0.098**	74.035	-33.893	0.173
					0.052	**0.928**	**0.095**	74.354	-34.052	0.147
				-0.037		**0.927**	**0.093**	74.954	-34.352	0.109
	-0.024					**0.927**	**0.092**	75.17	-34.46	0.098
		0.018				**0.927**	**0.093**	75.431	-34.59	0.086
			-0.046	-0.031		**0.927**	**0.097**	75.726	-33.653	0.074
		-0.035	-0.076			**0.929**	**0.101**	75.775	-33.677	0.072
				-0.029	0.047	**0.927**	**0.094**	76.111	-33.845	0.061
	-0.009		-0.045			**0.927**	**0.097**	76.15	-33.864	0.06
			-0.039		0.014	**0.927**	**0.097**	76.174	-33.876	0.059
	-0.019				0.049	**0.927**	**0.095**	76.199	-33.889	0.059

Abreviations: AICc, second order AIC; w_i, Akaike weight of each model. Significant values of parameters estimates and of Moran's I are in bold.

Figure S1 Eigenvalues of sPCA for cohorts 1 and 2. A) Eigenvalues decomposition into the genetic variance explained and Moran's I values. B) Barplot of eigenvalues showing positive (global structures) and negative (local structures) axes. In both, the selected sPCs for the spatial analyses are highlighted.

A

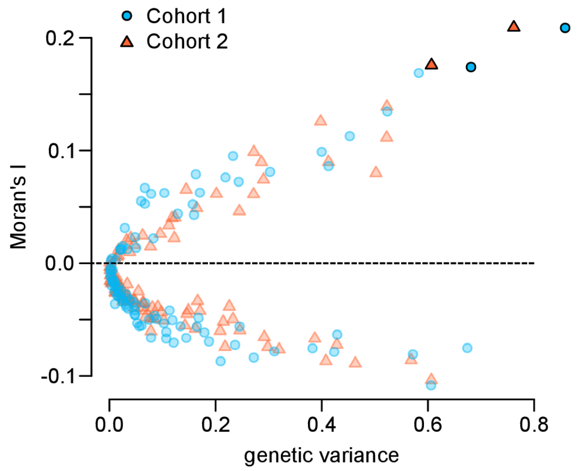

B

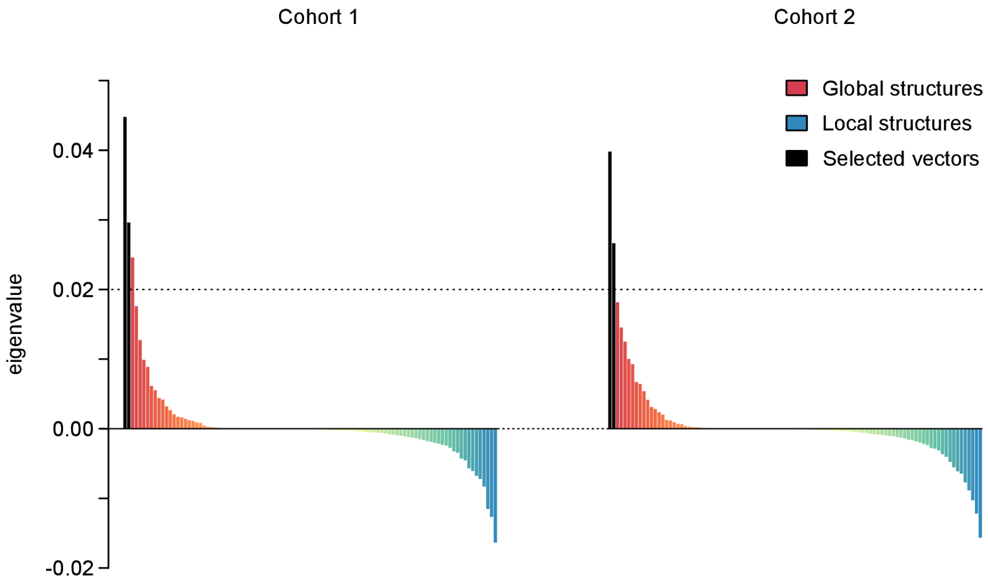

Figure S2 Lagged principal scores and inferred values of environmental variables. A) PC1 and anions. B) PC2 and cations 1.

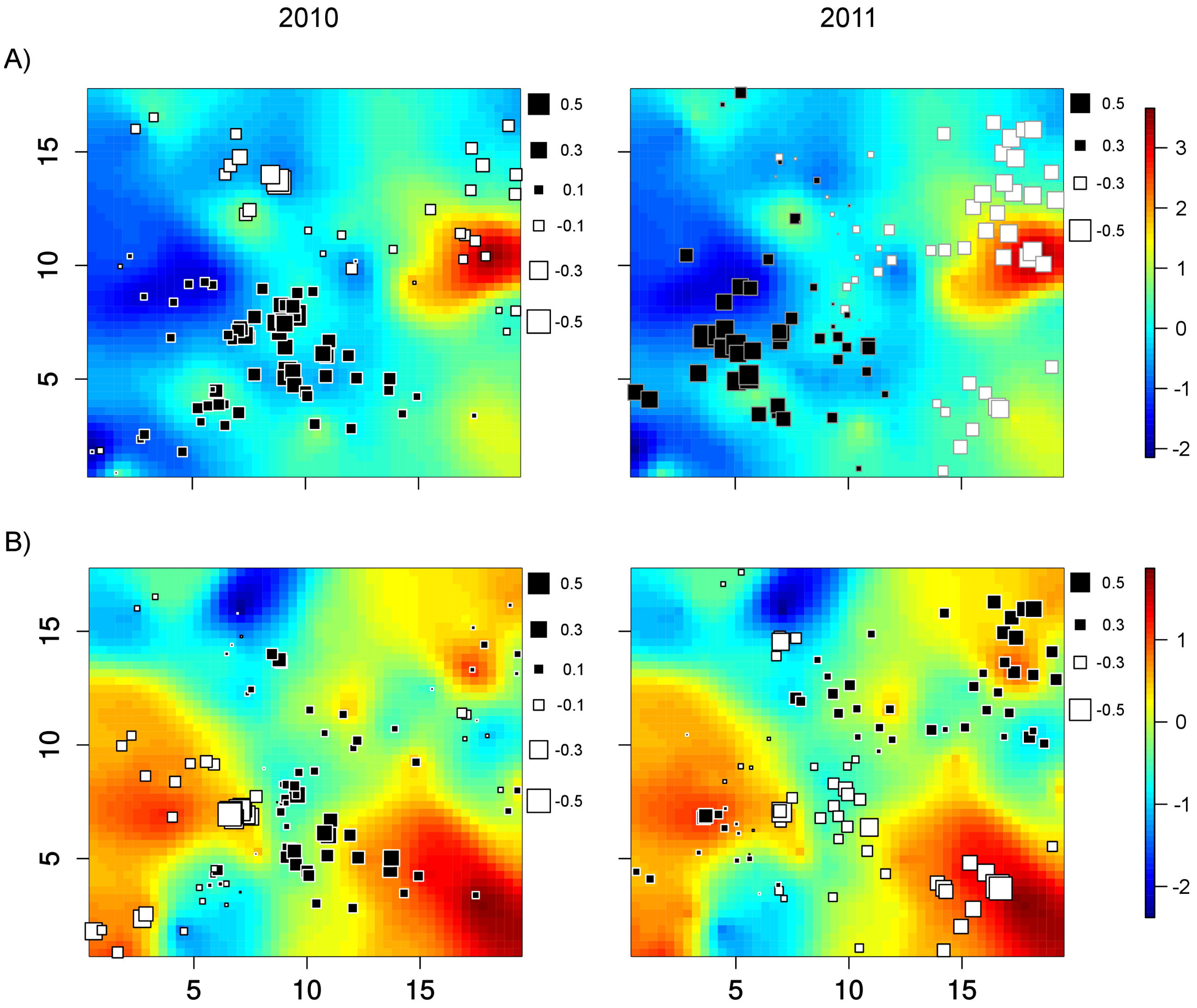

Figure S3 Ripley's K functions for both cohorts

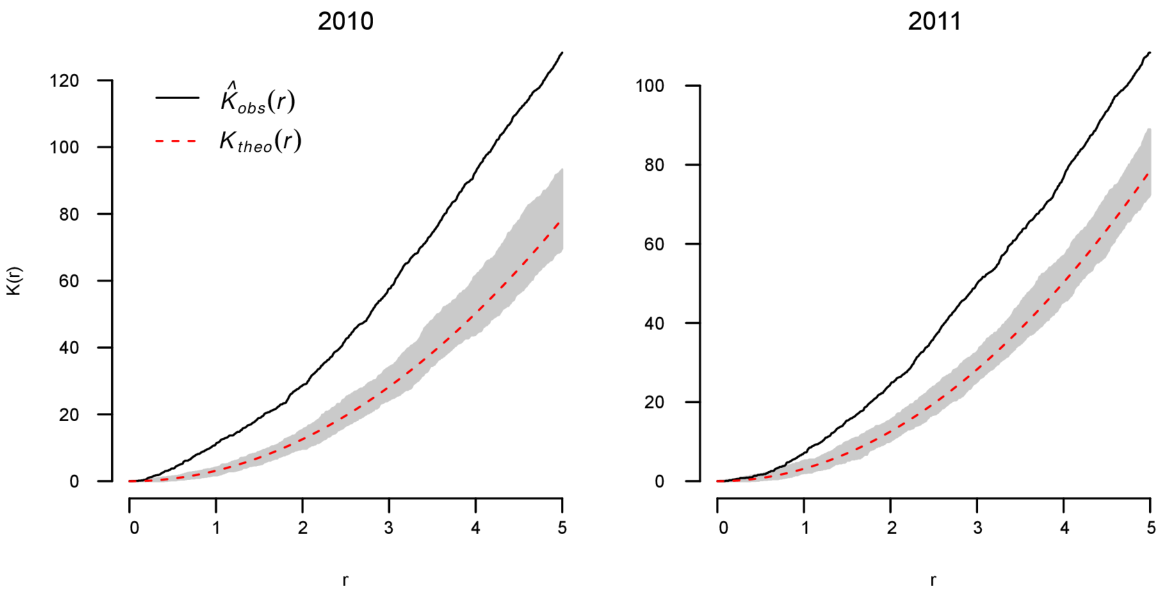

Figure S4 Pathways of inter-annual migration. Life cycle of two flowering cohorts (cohort 1 and cohort 2) are shown along four years (year 1 to 4). Dashed lines define the different hypothetical pathways taken by an inter-annual migrant from the flowering season of the cohort 1 to that of cohort 2.

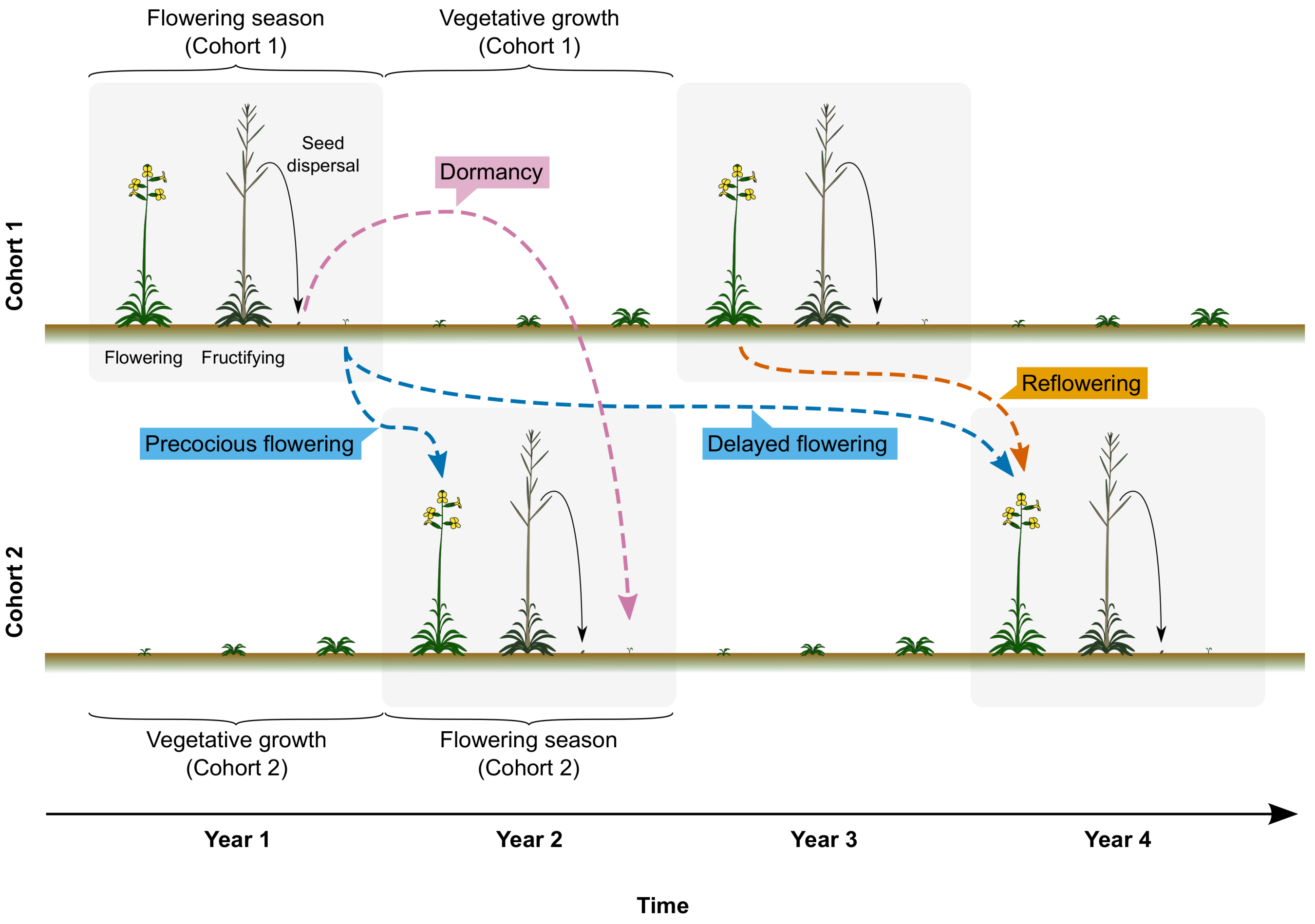

Figure S5 Biplot of the PCA performed over the soil cations. Circles and triangles depict the scores of the observations on the principal components 1 and 2 (cations 1 and cations 2). The coefficients of the variables are marked with arrows.

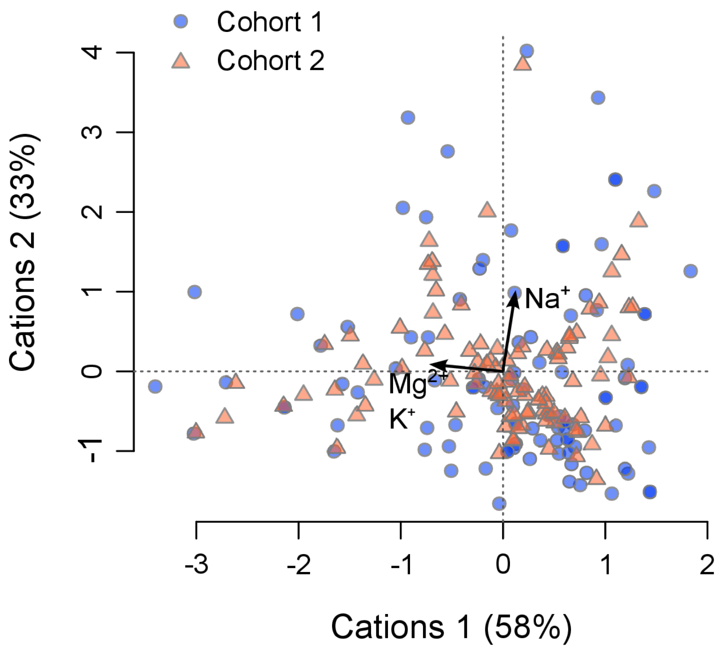

Supplement: Supplementary Information [file srep37712-s1.pdf]
